# Revision of *Paranastatus* Masi (Eupelmidae, Eupelminae) with descriptions of four new species

**DOI:** 10.3897/zookeys.559.6134

**Published:** 2016-02-03

**Authors:** Melanie L. Scallion, Gary A.P. Gibson, Barbara J. Sharanowski

**Affiliations:** 1 University of Manitoba, 214 Animal Science Building, Winnipeg, Manitoba, Canada R3T 2N2; 2 Canadian National Collection of Insects, Arachnids and Nematodes, Agriculture and Agri-Food Canada, Ottawa, Ontario, Canada K1A 0C6

**Keywords:** Graeffea crouanii, Anastatus, South Pacific, Indian Ocean, dispersal mechanisms, biodiversity, lectotye designation, identification key

## Abstract

*Paranastatus* Masi, 1917 (Eupelmidae, Eupelminae) was originally described based on two species from Seychelles: *Paranastatus egregius* and *Paranastatus violaceus*. Eady (1956) subsequently described *Paranastatus nigriscutellatus* and *Paranastatus verticalis* from Fiji. Here, four new species of *Paranastatus* are described: *Paranastatus bellus* Scallion, **sp. n.** and *Paranastatus pilosus* Scallion, **sp. n.** from Indonesia, and *Paranastatus halko* Scallion, **sp. n.** and *Paranastatus parkeri* Scallion, **sp. n.** from Fiji. A key to all *Paranastatus* species based on females is included and lectotypes are designated for *Paranastatus egregius* and *Paranastatus violaceus*. Finally, previously unobserved colour variation from newly collected material of *Paranastatus verticalis*, distribution patterns of species, and possibilities for future research are discussed.

## Introduction

*Paranastatus* Masi, 1917 (Eupelmidae, Eupelminae) is one of 33 currently recognized genera within Eupelminae (Gibson 1995). This genus was initially established for two species based primarily on the distinctive triangular shape of the head of females. Four species have been described to date: *Paranastatus egregius* Masi, 1917 and *Paranastatus violaceus* Masi, 1917 from Seychelles, and *Paranastatus verticalis* Eady, 1956 and *Paranastatus nigriscutellatus* Eady, 1956 from Fiji (Masi 1917, Eady 1956). No new specimens of either *Paranastatus egregius* or *Paranastatus violaceus* have been reported since their original description and their biology remains unknown. However, O’Conner et al. (1955) and Rapp (1995) subsequently reared *Paranastatus nigriscutellatus* and *Paranastatus verticalis* from the eggs of the walking stick, *Graeffea crouanii* Le Guillou (Phasmatodea: Phasmatidae). Males are known only for *Paranastatus egregius*, *Paranastatus nigriscutellatus*, and *Paranastatus verticalis*. A key to these males was provided by Eady (1956).

Eupelmidae is likely a grade-level taxon (Gibson 1989) rather than being monophyletic (Heraty et al. 2013), though Eupelminae is supported as monophyletic (Gibson 1989). The subfamily is characterized in part by its extreme sexual dimorphism, and species and higher level taxonomy is based primarily on female morphology. Eupelmines are parasitoids or predators of eggs and primary or hyperparasitoids of other immature stages of various arthropods, including Blattaria, Diptera, Hemiptera, Hymenoptera, Lepidoptera, Mantodea, Orthoptera and Phasmida, as well as Araneae and even Pseudoscorpionida (Gibson 1995, Austin et al. 1998). Gibson (1995) hypothesized that *Paranastatus* and five other genera, including *Anastatus* Motschulsky, formed a monophyletic clade, though with unresolved relationships and with *Paranastatus* possibly rendering *Anastatus* paraphyletic. Like known *Paranastatus*, members of *Anastatus* are mostly egg parasitoids and have been recorded as endoparasitoids of the eggs of Phasmida (Gibson 1995).

More recent collections from the South Pacific revealed new specimens of *Paranastatus*, including what appeared to be undescribed species. The purpose of this study was to differentiate and describe these new species and provide observations on variation observed among new *Paranastatus verticalis* material. An illustrated key to the world species of female *Paranastatus* is also included.

## Methods

Type material of *Paranastatus verticalis*, *Paranastatus nigriscutellatus*, *Paranastatus egregius*, and *Paranastatus violaceus* was examined as part of a loan from The Natural History Museum, London, England (BMNH). Paratypes of one female of *Paranastatus nigriscutellatus* and a male and female of *Paranastatus verticalis* were also examined on loan from the U.S. National Museum of Natural History, Washington, DC, USA (USNM). Other material was borrowed from the Canadian National Collection of Insects, Arachnids and Nematodes, Ottawa, ON, Canada (CNC). Some of the latter material was collected in projects requiring primary type material to be returned to the Bernice P. Bishop Museum, Honolulu, HI, USA (BPBM).

Two systems were used to image specimens, a Nikon D5200 camera mounted on an Olympus SZX16 stereomicroscope, and a Canon DSLR 7D Mark II camera with a MP-E 65mm macro lens attached to a motorized rail. Images were taken at multiple levels of focus, and stacked into a single image using the program Helicon Focus 6 (Helicon Soft Ltd, 2014). Images were processed and enhanced using Adobe Photoshop CC 2014. Scanning electron microscope images were obtained using a Hitachi Tabletop Microscope TM-1000. Measurements were taken using a Motic SMZ-168 microscope with an Olympus G10x micrometre eyepiece. Body length was measured in millimetres a total of three times and averaged. Excluding primary types, imaged specimens are labelled with a “JBWM Photo 2015-X” specimen number label. This is cited with other label data given for the respective specimen, in the Suppl. material 1: “*Paranastatus* Label Data”, and in the figure captions.

Structure and sculpture terminology follows Gibson et al. (1997), but additional clarification on sculpture terminology is provided below. Images are provided for clarity where necessary in the keys and descriptions. Alutaceous and coriaceous are similar in that they both mean leather-like (Harris 1979). Here, alutaceous refers to sculpturing where fine grooves create elongated cells, whereas coriaceous refers to sculpturing where the fine grooves create more square but irregularly-shaped cells. Coriaceous-imbricate refers to cells that appear overlapping. Reticulate refers to cells that are delineated by ridges. Pustulate refers to a bumpy texture, whereas granulate refers to many fine bumps, like granules. Rugose means wrinkled, whereas rugulose means very finely wrinkled.

Facial structuring can be divided into the lower face (region below toruli to clypeus and between malar sulci), scrobes (depressions rising above toruli and joining anterior to frontovertex), and interantennal area (region between scrobes and toruli). Gena refers to the region delineated by the malar sulcus and occipital margin, and extends to halfway along posterior margin of the eye. The vertex lies between the eyes from the frontovertex to the posterior margin of the eyes, where the temple begins. The temple runs between the posterior occipital margin and eyes to the genae.

Colouration of the antennomeres is often a quick identifier to species because females of four species have unique antennal colouration, though females of two species have overlapping colour patterns and two have the same colour pattern. The sculpture of the mesoscutum in combination with the extent of its concavity can be used to separate species with similar antennal colouration.

Due to the number of specimens examined, paratype and other material label data has been condensed for a few species to include localities, dates collected, and collector. A number in brackets corresponds to the number of specimens from each locality. For verbatim label data, see the Suppl. material 1: “*Paranastatus* Label Data”. A double line, ǁ, represents a new line on the label, and ++ represents a separate label.

## Taxonomy

For a key to the world species of known *Paranastatus* males (*Paranastatus egregius*, *Paranastatus nigriscutellatus*, and *Paranastatus verticalis*), see Eady (1956).

### Key to world species of *Paranastatus* Masi based on females

Note: When viewing mandible dentition, a dorsolateral view with the teeth directed forward is best for visualizing dentition (see Fig. 3).

**Table:** 

1	Mandible tridentate (Fig. 1). Flagellum mostly white or, if mostly brown, then flagellomere 7 entirely brown or white only apically. Lower face reticulate (Fig. 1). Gena mostly reticulate, or coriaceous to coriaceous-imbricate along occipital margin toward temple (Fig. 2)	**2**
–	Mandible quadridentate (Fig. 3). Flagellum mostly brown basally but flagellomeres 7 and 8 entirely white or light yellow-brown and sometimes flagellomere 6 white. Lower face smooth to alutaceous or coriaceous (Fig. 3). Gena alutaceous or coriaceous (Fig. 4)	**5**
2(1)	Head in lateral view with vertex raised between eyes, and temple flat such that temple and occiput at almost a right angle (Fig. 5). Temple smooth. Lower face with fringe of setae below toruli (Fig. 6). Flagellum brown except flagellomere 8 and club white (Figs 15, 17)	***Paranastatus verticalis* Eady**
–	Head in lateral view with vertex and temple slightly to distinctly convex between eyes, and temple and occiput at an obtuse angle (Figs 7, 8). Temple variably sculptured. Lower face with setae, but not arranged as a fringe (Figs 1, 3). Flagellum colour variable	**3**
3(2)	Vertex smooth posterior to ocelli towards temple. Antenna with scape blue (Fig. 8) and flagellum brown except flagellomere 8, club, and usually apex of flagellomere 7 white (Fig. 19). Mesoscutum smooth to slightly rugulose. Gaster with tergites brown except apex of gaster green; sternites brown except white at very base	***Paranastatus halko* Scallion, sp. n.**
–	Vertex rugose or reticulate posterior to ocelli towards temple. Antenna mostly white except basal half of scape brown and club variable in colour. Mesoscutum reticulate (Fig. 9). Gaster brown except tergites 1 and 2 white; sternites variable	**4**
4(3)	Vertex rugose posterior to ocelli (Fig. 10). Temple coriaceous and brownish-green to blue-green laterally. Gena blue-green, coriaceous to reticulate along malar sulcus. Antennal club brown. Mesoscutum purple-brown medially, straw yellow laterally, and reticulate. Fore wing hyaline behind distal half of submarginal vein, but deeply infuscate basally, lightly infuscate (tinted brown) in patch behind base of marginal vein, and infuscate from behind postmarginal vein to wing apex. Gaster brown beyond basal white sternites	***Paranastatus bellus* Scallion, sp. n.**
–	Vertex reticulate posterior to ocelli (Fig. 11). Temple reticulate and dark blue-purple. Gena blue-purple, reticulate. Antennal club white except slightly darkened apically. Mesoscutum blue-purple medially, brown laterally, and reticulate. Fore wing hyaline except lightly infuscate in apical half. Gaster purplish-brown beyond basal white sternites	***Paranastatus pilosus* Scallion, sp. n.**
5(1)	Temple smooth, and in dorsal view occipital margin straight. Flagellum brown except flagellomeres 7, 8 and club white. Pronotum smooth dorsally or coriaceous only along anterior edge. Mesoscutum distinctly and deeply concave posteromedially (Fig. 12). Fore wing infuscate with hyaline band behind distal half of submarginal vein	**6**
–	Temple coriaceous or pustulate, and in dorsal view occipital margin concave. Flagellum variable in colour, but club brown. Pronotum coriaceous dorsally. Mesoscutum slightly concave posteromedially (Fig. 13). Fore wing variable	**7**
6(5)	Head with face green to coppery-green, lower face alutaceous to coriaceous centrally (Fig. 3), and interantennal area and scrobes coriaceous. Frontovertex with blunt teeth projecting towards vertex. Mesoscutum mostly alutaceous, except coriaceous posteromedially (Fig. 12). Legs with profemur black-brown except for light brown patch ventroapically, mesofemur black-brown dorsally and yellow ventrally and basally, and metafemur yellow basally and darkening to brown apically. Gaster brown except green apically, basal tergites white centrally and sternites 1–4 white	***Paranastatus nigriscutellatus* Eady**
–	Head with face dark purple-brown and entirely smooth to alutaceous. Frontovertex smooth with a few small bumps. Mesoscutum smooth, except slightly coriaceous posteromedially. Legs with all femora straw yellow. Gaster green apically, but tergites otherwise dark coppery-green and sternites brown	***Paranastatus parkeri* Scallion, sp. n.**
7(5)	Vertex coriaceous and dull black-brown. Flagellum with apical two funiculars light yellow-brown. Mesoscutum dark purple-brown, and mostly alutaceous except coriaceous posteromedially (Fig. 13). Fore wing evenly infuscate. Gaster dark brown except apical tergites green and sternites 1 and 2 light brown	***Paranastatus violaceus* Masi**
–	Vertex pustulate, purple except for coppery sheen between ocelli (Fig. 14). Flagellum with apical three funiculars white. Mesoscutum light brown, and slightly coriaceous. Fore wing with hyaline band behind distal half of submarginal vein, lightly infuscate behind base of marginal vein and behind postmarginal vein, hyaline between infuscate regions and apically (Fig. 27). Gaster dark brown except tergites 1 and 2 and sternites 1–3 white	***Paranastatus egregius* Masi**

**Figures 1–8.**
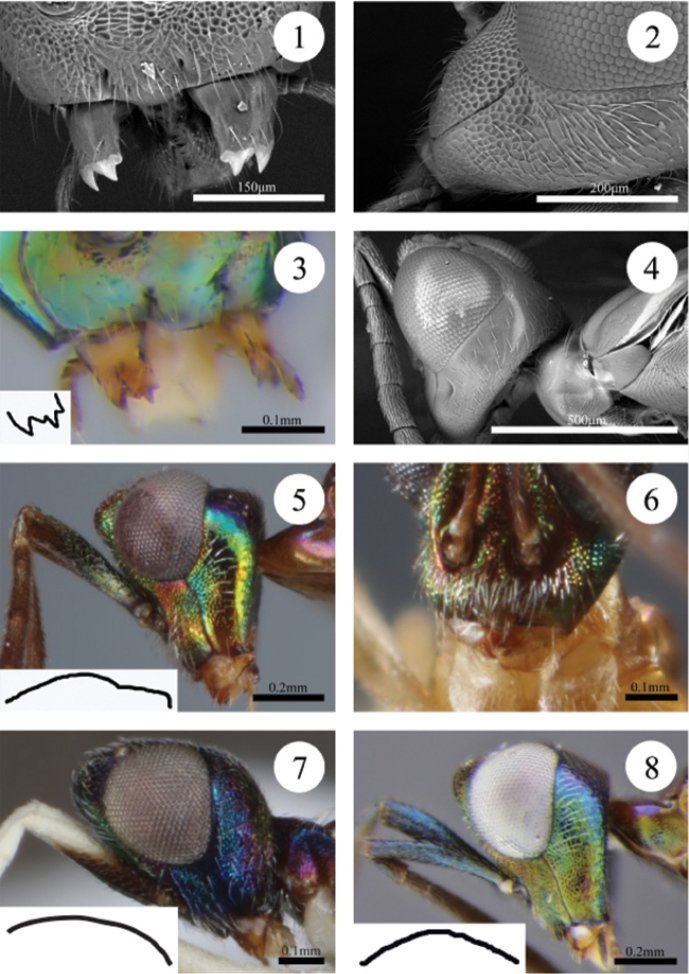
**1** Scanning electron micrograph (SEM) of *Paranastatus halko*, lower face in anterior view showing mandibular dentition (JBWM Photo 2015-01) **2** SEM of *Paranastatus halko*, gena in lateral view (JBWM Photo 2015-02) **3** *Paranastatus nigriscutellatus*, lower face in frontolateral view showing mandible dentition (JBWM Photo 2015-03). Inset: outline of mandible dentition showing four teeth **4** SEM of *Paranastatus nigriscutellatus*, head and anterior part of mesosoma in lateral view (JBWM Photo 2015-03) **5** *Paranastatus verticalis*, head in lateral view. Inset: outline of vertex-temple shape (JBWM Photo 2015-04) **6** *Paranastatus verticalis* holotype, lower face in anterior view showing fringe of setae below toruli **7** *Paranastatus pilosus*, head in lateral view. Inset: outline of vertex-temple shape (JBWM Photo 2015-05) **8** *Paranastatus halko* holotype, head in lateral view. Inset: outline of vertex-temple shape.

**Figures 9–14.**
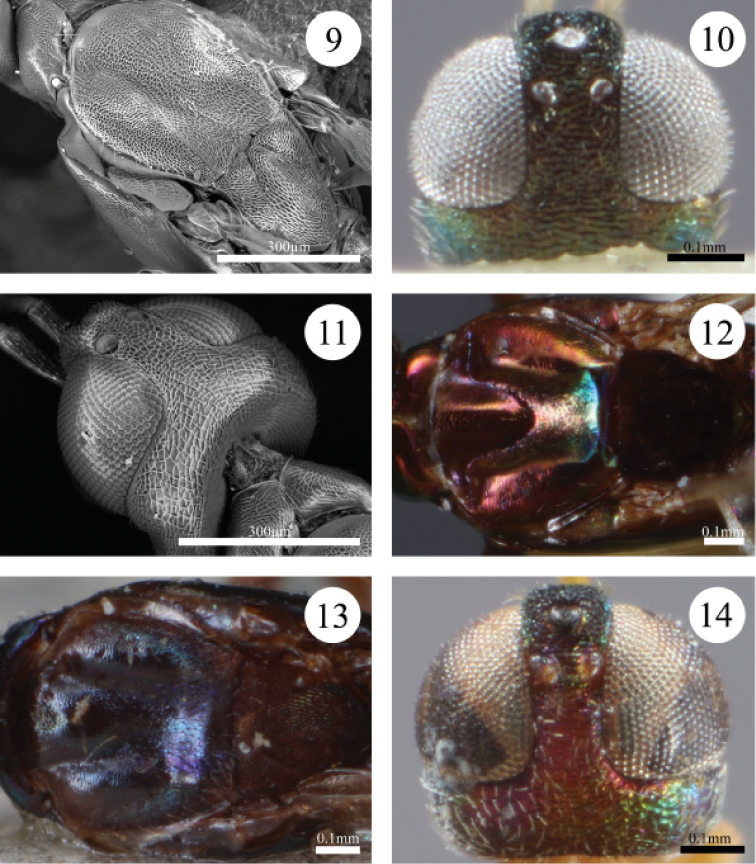
**9** Scanning electron micrograph (SEM) of *Paranastatus pilosus*, mesonotum in dorsal view (JBWM Photo 2015-05) **10** *Paranastatus bellus* holotype, head in dorsal view **11** SEM of *Paranastatus pilosus*, head and pronotum in dorsal view (JBWM Photo 2015-05) **12** *Paranastatus nigriscutellatus* holotype, mesonotum in dorsal view **13** *Paranastatus violaceus* lectotype, mesonotum in dorsal view **14** *Paranastatus egregius* lectotype, head in dorsal view.

**Figures 15–21.**
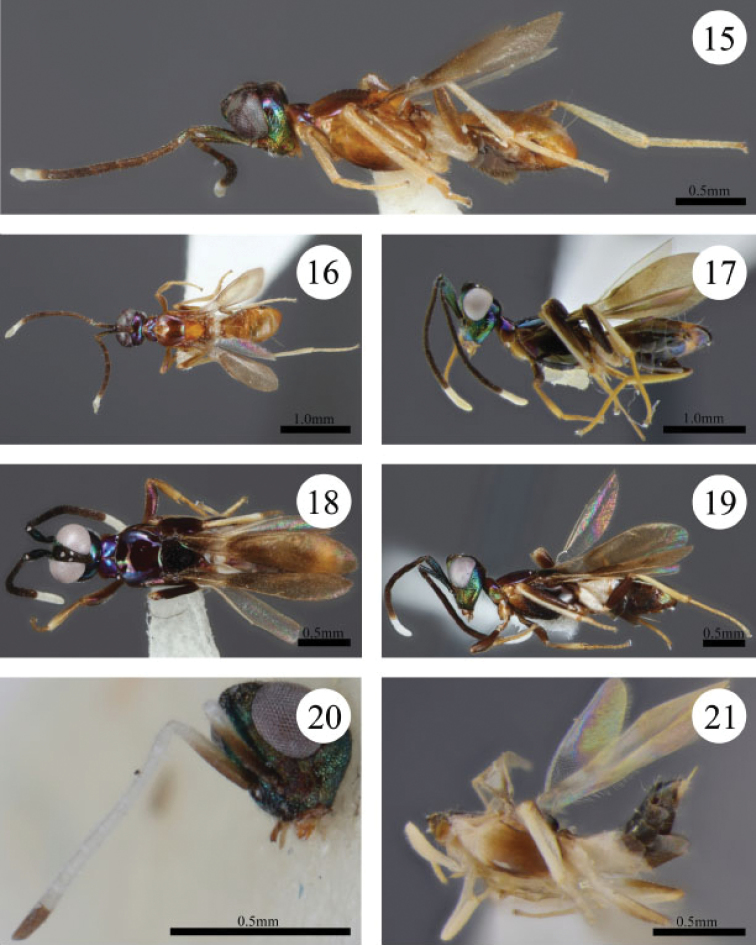
**15** *Paranastatus verticalis* holotype, lateral habitus **16** *Paranastatus verticalis* holotype, dorsal view **17** *Paranastatus verticalis* (Taveuni, Fiji), lateral habitus (JBWM Photo 2015-06) **18** *Paranastatus verticalis* (Taveuni, Fiji) in dorsal view (JBWM Photo 2015-06) **19** *Paranastatus halko* holotype, lateral habitus **20** *Paranastatus bellus* holotype, head in frontolateral view and antenna **21** *Paranastatus bellus* holotype, body in lateral view.

**Figures 22–27.**
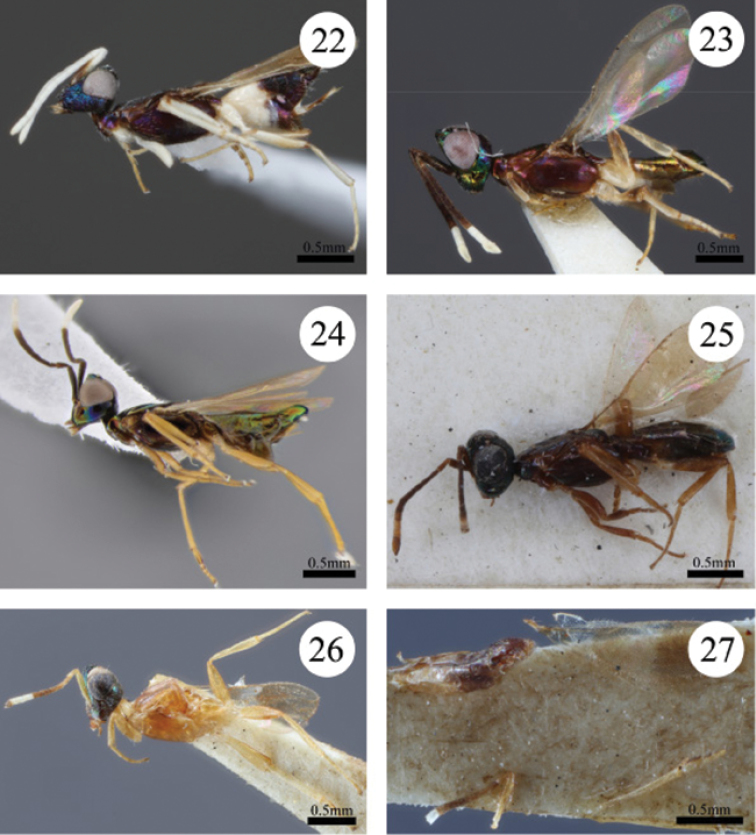
**22** *Paranastatus pilosus* holotype, lateral habitus **23** *Paranastatus nigriscutellatus* holotype, lateral habitus **24** *Paranastatus parkeri* holotype, lateral habitus **25** *Paranastatus violaceus* lectotype, lateral habitus **26** *Paranastatus egregius* lectotype, partial body in lateral view **27** *Paranastatus egregius* lectotype broken body parts glued to the point.

### Paranastatus bellus

Taxon classificationAnimaliaHymenopteraEupelmidae

Scallion sp. n.

http://zoobank.org/65D79CA1-0DA8-4483-9884-9F3694D5ED5B

Figs 10 , 20 , 21

#### Material examined.

Holotype female, dry pinned, deposited in BMNH (Hym Type 5.4813, barcode NHMUK010198566). Label data: “SULAWESI UTARA: Dumoga-Bone Nat. Pk, edge of rainforest, 0°34'N, 123°54'E. A.D. Austin June 1985, M.T.”

Paratype female, dry pinned, deposited in CNC. Label data: “INDONESIA. Sulawesi Utara, Dumoga Bone Nat. Pk, Toraut IV.1985, JS Noyes, forest edge, MT.”

#### Diagnosis.

Females of *Paranastatus bellus* are differentiated by the following combination of features: vertex rugose (Fig. 10); antenna mostly white except base of scape and club brown (Fig. 20); mandible tridentate; mesoscutum purple-brown medially, straw-yellow laterally and reticulate.

#### Description.

**Female.** Length: 2 mm.

*Colour*. Head with vertex dull black-brown (Fig. 10); temple brownish-green dorsally, blue-green laterally (Fig. 10); gena and face metallic blue-green (Fig. 20); frontovertex dull black-brown. Antenna mostly white, except base of scape and club brown (Fig. 20). Pronotum light brown; mesoscutum purple-brown medially, straw-yellow laterally; scutellar-axillar complex dark orange-brown; mesopleuron light brown to white anteriorly (Fig. 21). Legs white with mesofemur darkened along posterior apical edge and metafemur darkening to brown apically. Fore wing hyaline behind distal half of submarginal vein, but deeply infuscate basally, lightly infuscate patch behind base of marginal vein, and infuscate from behind postmarginal vein to wing apex; hind wing hyaline. Gastral tergites 1 and 2 white, remaining tergites dark brown; gastral sternites 1–4 white, remainder brown. Colour of setae on various body regions discussed in appropriate sections below.

*Head*. Vertex rugose (Fig. 10); temple coriaceous (Fig. 10); gena coriaceous except reticulate along malar sulcus (Fig. 20); face reticulate; frontovertex with blunt teeth projecting posteriorly towards vertex. Mandible tridentate. Vertex, temple and gena with sparse, very light brown setae; face with sparse white setae except scrobes bare; eyes with dense, short white setae.

*Mesosoma*. Pronotum coriaceous; mesoscutum reticulate, distinctly concave posteromedially; scutellar-axillar complex reticulate; mesopleuron coriaceous. Pronotum with white setae, setae longer along posterior edge; mesoscutum with many white setae; scutellar-axillar complex with few white setae along edges; mesopleuron with white setae anteriorly, remainder bare. Fore wing with dense, short brown setae; hind wing with relatively fewer short, light brown setae.

*Metasoma*. Entirely coriaceous with white setae ventrally, the setae very sparse dorsally and long at apex of gaster.

**Male.** Unknown.

#### Etymology.

From the Latin *bellus*, meaning handsome, in memory of Melanie Scallion’s dog Beau (French: handsome). This is an adjective in the nominative singular.

#### Distribution.

Sulawesi Island, Indonesia.

#### Biology.

Unknown.

#### Remarks.

Holotype deposited in the BMNH at the request of Dr. Andrew Austin, University of Adelaide, Australia. Both specimens are in poor condition. The head of the holotype is glued to the point, and the paratype is contorted with the body curled up on itself.

### Paranastatus egregius

Taxon classificationAnimaliaHymenopteraEupelmidae

Masi, 1917

Figs 14 , 26 , 27

Paranastatus egregius Masi, 1917: 165–166.

#### Material examined.

Lectotype female, here designated; dry pinned, deposited in BMNH (Hym Type 5.1,035a). Label data: “Mahe, ’08–9 Seychelles Exp. Percy Sladen Trust Exped. B.M. 1913-170.”

Paralectotype male, here designated; dry pinned, deposited in BMNH. Label data: “Mahe, ’08–9 Seychelles Exp. Percy Sladen Trust Exped. B.M. 1913-170.”

#### Diagnosis.

Females of *Paranastatus egregius* are differentiated by the following combination of features: vertex behind ocelli and temple pustulate (Fig. 14); antenna brown except flagellomeres 6–8 white (Fig. 26); mandible quadridentate; mesoscutum light brown, slightly coriaceous and only slightly concave posteromedially. Males of *Paranastatus egregius* are differentiated by the following combination of features: vertex weakly reticulate; mandible bidentate; mesoscutum convex; colouration similar to females.

#### Distribution.

Mahé Island, Seychelles.

#### Biology.

Unknown.

#### Remarks.

Masi (1917) established *Paranastatus egregius* based on one female and two males, but without designating a holotype. Of the three specimens, the BMNH only has the female and one of the males in its collection (Dale-Skey, pers. comm.). Here we designate the female as lectotype and the male as paralectotype, and have labelled the specimens accordingly. The location of the second male is presently unknown.

### Paranastatus halko

Taxon classificationAnimaliaHymenopteraEupelmidae

Scallion sp. n.

http://zoobank.org/6881140A-142F-48F1-89A6-9748FB3361FE

Figs 1 , 2 , 8 , 19

#### Material examined.

Holotype female, dry pinned, deposited in BPBM (Type No. 17540). Label data: “FIJI: Viti Levu, Vuda Prov., Koroyanitu Pk, 1 km E Abaca Vlg., Savuione Trl, 800m, 22.IV–6.V.03 Malaise 1, Schlinger, Tokota’a. 17.667°S, 177.55°E. FBA 180165.”

Paratype females (24), dry pinned, deposited in BPBM and CNC. Collecting data for all specimens examined are listed below. However, date ranges are provided when multiple specimens were collected from the same locality with the full label data for each specimen listed in Suppl. material 1: “*Paranastatus* Label Data”.

(14). **FIJI**. Viti Levu, Vuda Prov., 1 km E Abaca Vlg., Koroyanitu Ntl. Pk, Savuione Trl. Dates collected range from 7.X.2002–6.V.2003 by E. Schlinger and M. Tokota’a.

(4, includes JBWM Photo 2015-02). **FIJI**. Viti Levu, Vuda Prov., 0.5 km N Abaca Vlg., Koroyanitu Eco Pk, Mt Evan’s Range. Dates collected range from 26.X–3.XII.2002 by E. Schlinger and M. Tokota’a.

(2). **FIJI**. Viti Levu, Naitasiri Prov., 4 km WSW Colo-i-Suva Vlg., Mt Nakobalevu, 300m. Collected 12.IV.2004 by E. Schlinger and M. Tokota’a.

(4, includes JBWM Photo 2015-01). **FIJI**. Viti Levu, Naitasiri Prov., 4 km WSW Colo-i-Suva Vlg., Mt Nakobalevu, 372m. Dates collected range from 25.II–14.XI.2003 by E. Schlinger and M. Tokota’a.

#### Diagnosis.

Females of *Paranastatus halko* are differentiated by the following combination of features: vertex granulate between ocelli and smooth posterior to ocelli; temple smooth; scape and pedicel blue (Fig. 8); mandible tridentate (Fig. 1); mesoscutum smooth or very slightly rugulose.

#### Description.

**Female.** Length: 2.6–2.95 mm.

*Colour*. Head with vertex dull black-brown between ocelli and metallic green changing to blue-purple posterior to ocelli towards temple; temple shining metallic blue-purple; gena and face metallic coppery-green (Figs 8, 19); frontovertex usually brown, sometimes blue-green with brown centrally (5/25 specimens). Antenna with scape and pedicel blue (Fig. 8), anellus (flagellomere 1) and flagellomeres 2–6 brown, tip of 7 usually white but sometimes brown, 8 and club all white (Fig. 19). Pronotum metallic blue-purple dorsally, coppery-green laterally; mesoscutum reddish-brown to more orange-brown; scutellar-axillar complex dull black; mesopleuron brown. Legs with procoxa light yellow-brown although sometimes dark brown (3/25 specimens), protrochanter dark brown, sometimes light yellow-brown (8/25), and profemur dark black-brown, sometimes with light spot apically on ventral surface (1/25); mesocoxa light yellow-brown, mesotrochanter light brown, mesofemur black-brown dorsally with a lighter streak ventrally, and mesotibia white; metacoxa white, sometimes with brown spot basoventrally (1/25), metatrochanter light brown, sometimes white (2/25), and metafemora dark black-brown; remaining tibiae and tarsomeres light yellow-brown to straw-yellow. Fore wing infuscate with hyaline band behind distal half of submarginal vein; hind wing hyaline. Gastral tergites brown, apex of gaster green; sternites brown except white at very base. Colour of setae on various body regions discussed in appropriate sections below.

*Head*. In lateral view, vertex distinctly convex between eyes, and temple sloping toward occiput to create a strongly obtuse angle (Fig. 8); vertex granulate between ocelli and smooth posterior to ocelli, sometimes appearing pustulate due to setae; temple smooth; gena coriaceous-imbricate to reticulate along malar sulcus (Figs 2, 8); face reticulate; frontovertex usually with blunt teeth projecting posteriorly towards vertex. Mandible tridentate, possibly appearing quadridentate in some views due to slight bump on ventral edge of large middle tooth (Fig. 1). Vertex and temple with evenly dispersed light brown setae; gena with brown setae, but with a patch of thick white setae on upper part of gena below eye; parascrobal region and region between toruli and clypeus with thick white setae; eye with short white setae; face otherwise with thinner brown setae.

*Mesosoma*. Pronotum coriaceous; mesoscutum usually smooth, sometimes very slightly rugulose, and only slightly concave posteromedially; scutellar-axillar complex reticulate; mesopleuron coriaceous. Pronotum with few brown setae; mesoscutum with sparse light brown setae posteromedially and along margins; scutellar-axillar complex with few light brown setae; mesopleuron bare. Fore wing with dense, short brown setae; hind wing with relatively fewer short, light brown setae.

*Metasoma*. Entirely coriaceous; short to long brown setae evenly distributed ventrally; very sparse, short brown setae dorsally.

**Male.** Unknown.

#### Etymology.

Named in honour of Ed and Eliz Halko from Winnipeg, Manitoba, Canada. Their daughter, Gail Halko, also from Winnipeg, has made a donation to the Wallis-Roughley Museum of Entomology at the University of Manitoba to honour her parents, who both celebrated their 85^th^ birthdays in 2015. This is a noun in apposition to retain integrity of the name Halko in the species name.

#### Distribution.

Viti Levu, Fiji.

#### Biology.

Unknown.

#### Remarks.

Vertex may appear pustulate under a stereomicroscope due to the setae. Care should be taken when using antennal colouration as a guide to species since flagellomere 7 is sometimes completely brown instead of white at apex, thus resembling the antennae of *Paranastatus verticalis*.

### Paranastatus nigriscutellatus

Taxon classificationAnimaliaHymenopteraEupelmidae

Eady, 1956

Figs 3 , 4 , 12 , 23

Paranastatus nigriscutellatus Eady, 1956: 61–64.

#### Material examined.

Holotype female, dry pinned, deposited in BMNH (Hym Type 5.1624a). Label data: “HY 976 FIJI Savu Savu II.1954 B.A. O’Connor. Ex *Graeffea crouani* eggs. Com. Inst. Ent Coll. No. 13599.”

Allotype male, dry pinned, deposited in BMNH. Label data: “HY 976 FIJI Savu Savu II.1954 B.A. O’Connor. Ex *Graeffea crouani* eggs. Com. Inst. Ent Coll. No. 13599.”

Paratype females (6), dry pinned.

(2, deposited in BMNH). HY 975 **FIJI** Taveuni XI.1953 B.A. O’Connor Ex eggs of *Graeffea crouani* in coconut crowns.

(3, deposited in BMNH). HY 976 **FIJI** Savu Savu II.1954 B.A. O’Connor Ex *Graeffea crouani* eggs.

(1, deposited in USNM). HY 975 **FIJI** Taveuni XI.1953 B.A. O’Connor Ex eggs of *Graeffea crouani* in coconut crowns.

#### Other material.

Females (11), dry pinned, deposited in BPBM and CNC. Collecting data for all specimens examined are listed below. However, date ranges are provided when multiple specimens were collected from the same locality with the full label data for each specimen listed in Suppl. material 1: “*Paranastatus* Label Data”.

(5). **FIJI**, Viti Levu, Namosi Prov., 2 km SE Nabukavesi Vlg., Ocean Pacific Rsrt, 40m. Dates collected range from 13.III–11.XII.2003 by E. Schlinger, M. Tokota’a and W. Naisilisili.

(2, includes JBWM Photo 2015-03). **FIJI**: Viti Levu, Vuda Prov., Koroyanitu Pk, 1 km E Abaca Vlg., SavuioneTrl, 800m. 22.IV–6.V.2003 by E. Schlinger and M. Tokota’a.

(1). **FIJI**: Kaduva I., 0.25 km SW Solodamu Vlg., Moanakaka Bird Sanctuary, 60m. Collected 9–30.V.2003 by E. Schlinger and M. Tokota’a.

(1). **FIJI**: Viti Levu, Sigatoka Prov., Sigatoka Sand Dunes Nat. Pk, 44m. Collected 12.II–12.III.2003 by E. Schlinger and M. Tokota’a.

(1). **FIJI**: Levu Is., Maitaesiri Prov., Hakobalevu Mt, 340m. Collected 12–24.III.2003 by M. Irwin et al.

(1). **FIJI**: Viti Levu, Naitasiri Prov., Bakobalevu logging rd. Collected 17.III–9.IV.2003 by E. Schlinger and M. Tokota’a.

#### Diagnosis.

Females of *Paranastatus nigriscutellatus* are differentiated by the following combination of features: face alutaceous to coriaceous centrally; mandible quadridentate (Fig. 3); pronotum smooth dorsally (sometimes coriaceous along anterior margin), alutaceous laterally; mesoscutum purplish-coppery, slightly bluish-green posteriorly, alutaceous to coriaceous posteromedially, distinctly concave posteromedially (Fig. 12). Males of *Paranastatus nigriscutellatus* are differentiated by the following combination of features: head entirely coriaceous; mandible quadridentate; scutellar-axillar complex coriaceous; colouration darker than that of females.

#### Distribution.

Islands of Fiji, Tonga, Western Samoa (Noyes 2015).

#### Biology.

Parasitoids of *Graeffea crouanii* eggs.

### Paranastatus parkeri

Taxon classificationAnimaliaHymenopteraEupelmidae

Scallion sp. n.

http://zoobank.org/536F9418-3E12-4D39-AD29-051A59D1FEAF

Fig. 24

#### Material examined.

Holotype female, dry pinned, deposited in BPBM (Type No. 17541). Label data: “FIJI: Viti Levu, 3.5 km N Veisari Stlmt, logging rd to Waivudawa, 14.II–8.III.03, 300m, Malaise 3, coll. E. Schlinger, M. Tokota’a 18.068°S, 178.367°E. FBA 136331.”

#### Diagnosis.

The unique female of *Paranastatus parkeri* is differentiated by the following combination of features: vertex and temple smooth; frontovertex smooth with a few small bumps; face smooth to alutaceous; mandible quadridentate; mesoscutum smooth except faintly coriaceous in posteromedial concavity.

#### Description.

**Female.** Length: 2.2 mm.

*Colour*. Head with vertex coppery between ocelli, metallic green to blue-purple posterior to ocelli; temple shining metallic green-purple dorsally to metallic blue-purple laterally; gena shining metallic coppery-green; entire face metallic dark purple-brown; frontovertex blue-green with brown centrally. Antenna with scape lightly shining green; pedicel, anellus (flagellomere 1), and flagellomeres 2–6 brown, 7, 8 and club white. Pronotum coppery-green; mesoscutum purplish-coppery, slightly bluish-green posteriorly; scutellar-axillar complex dull black; mesopleuron purple-coppery. Legs with procoxa dark brown, protrochanter light brown; mesocoxa light yellow-brown; metacoxa brown basally and white apically; remaining leg segments straw-yellow. Fore wing very lightly infuscate with hyaline band below distal half of submarginal vein; hind wing hyaline. Gaster green apically, tergites otherwise dark coppery-green and sternites brown. Colour of setae on various body regions discussed in appropriate sections below.

*Head*. Vertex and temple smooth; gena smooth to alutaceous along occipital margin; lower face smooth to alutaceous centrally, scrobes smooth to weakly alutaceous, interantennal area alutaceous; occipital margin straight in dorsal view; frontovertex smooth with a few small bumps. Mandible quadridentate. Entire head with sparse brown setae; eyes with sparse, very short white setae.

*Mesosoma*. Pronotum smooth; mesoscutum smooth to slightly coriaceous posteromedially, distinctly concave posteromedially; scutellar-axillar complex reticulate; mesopleuron coriaceous. Pronotum, mesoscutum, and scutellar-axillar complex with very few brown setae; mesopleuron with few short white setae anteriorly, remainder bares. Fore wing with dense, short brown setae; hind wing with relatively fewer short, brown setae.

*Metasoma*. Entirely coriaceous with long, brown setae sparsely distributed.

**Male.** Unknown.

#### Etymology.

Named in honour of Parker Brant, nephew of Barb Sharanowski, born November 2, 2012 in Australia to Julie and Billy Brant. This is a noun in the genitive case.

#### Distribution.

Viti Levu, Fiji.

#### Biology.

Unknown.

#### Remarks.

Abdomen was broken and lost after description and imaging had been completed. Antennae cannot be used as an identifying character in this species because the antennal colouration is the same as that of *Paranastatus nigriscutellatus*.

### Paranastatus pilosus

Taxon classificationAnimaliaHymenopteraEupelmidae

Scallion sp. n.

http://zoobank.org/57CABD16-BA30-4BDD-A5E8-74A0780A4A41

Figs 7 , 9 , 11 , 22

#### Material examined.

Holotype female, dry pinned, deposited in BMNH (Hym Type 5.4814, barcode NHMUK010198567). Label data: “INDONESIA: Seram, Solea VIII.1987, MT M. Day, forest.”

Paratype females (8), dry pinned, deposited in BMNH and CNC.

(4, includes JBWM Photo 2015-05). **INDONESIA**. Seram, Solea. IX.1987, M. Day.

(4). **INDONESIA**. Seram, Solea. VIII.1987, M. Day, forest.

#### Diagnosis.

Females of *Paranastatus pilosus* are differentiated by the following combination of features: vertex granulate between ocelli, reticulate posterior to ocelli (Fig. 11); temple reticulate (Fig. 11); antenna mostly white except scape brown basally and club lightly darkened apically (Fig. 22); mandible tridentate; mesoscutum blue-purple medially, brown laterally, and reticulate (Fig. 9).

#### Description.

**Female.** Length: 2.6 mm.

*Colour*. Head with vertex dull black-brown, sometimes purple-brown posterior to ocelli; temple dark blue-purple; gena blue-purple (Fig. 7); lower face mostly blue-purple but brown centrally below toruli; scrobes and interantennal area green or coppery-green; frontovertex dull black-brown or with blue centrally. Antenna white, except basal half of scape brown and very tip of club slightly darkened, and sometimes club completely white (Fig. 22). Pronotum metallic purple-blue, sometimes purple-brown laterally; mesoscutum blue-purple medially, brown laterally; scutellar-axillar complex dull black; mesopleuron purple. Legs with profemur white; mesofemur white with darkened posterior apical edge; metafemur white becoming yellow-brown apically; rest of legs white. Fore wing lightly infuscate in apical half, hyaline in basal half with small infuscate patch at base; hind wing hyaline. Gastral tergites 1–2 white, rest dark brown; gastral sternites 1–4 white, remainder purplish-brown. Colour of setae on various body regions discussed in appropriate sections below.

*Head*. Vertex granulate between ocelli, reticulate posterior to ocelli (Fig. 11); temple reticulate (Fig. 11); gena and face reticulate; occipital margin concave in dorsal view; frontovertex with blunt teeth projecting posteriorly towards vertex or sometimes granulate. Mandible tridentate. Head with white setae except scrobes bare; eyes with dense, short white setae.

*Mesosoma*. Pronotum coriaceous (Figs 9, 11); mesoscutum reticulate, distinctly concave posteromedially (Fig. 9); scutellar-axillar complex reticulate (Fig. 9); mesopleuron coriaceous. Pronotum with white setae, setae longer along posterior edge; mesoscutum with dense white setae; scutellar-axillar complex with few long white setae; mesopleuron with few white setae anteriorly, remainder bare. Fore wing with dense, short brown setae; hind wing with relatively fewer short, light brown setae.

*Metasoma*. Entirely coriaceous with white setae evenly distributed ventrally, setae sparser and shorter dorsally, and longer at apex of gaster.

**Male.** Unknown.

#### Etymology.

From Latin *pilosus*-hairy, in reference to the females having noticeably more setae than the other species. This is an adjective in the nominative case.

#### Distribution.

Seram Island, Indonesia.

#### Biology.

Unknown.

### Paranastatus verticalis

Taxon classificationAnimaliaHymenopteraEupelmidae

Eady, 1956

Figs 5 , 6 , 15–18

Paranastatus verticalis Eady, 1956: 64–65.

#### Material examined.

Holotype female, dry pinned, deposited in BMNH (Hym Type 5.1625a). Label data: “HWY 976 FIJI Suva Suva VI.1954 B.A. O’Connor. ex eggs of *Graeffea crouani* C.I.E.Coll. 13792.”

Allotype male, dry pinned, deposited in BMNH. Label data: “HWY 976 FIJI Suva Suva VI.1954 B.A. O’Connor. ex eggs of *Graeffea crouani* C.I.E.Coll. 13792.”

Paratype females (7), dry pinned.

(6, deposited in BMNH, includes JBWM Photo 2015-04). HY 976 **FIJI** Suva Suva VI.1954.

(1, deposited in USNM). HY 976 **FIJI** Suva Suva VI.1954 B.A. O’Connor.

#### Other material.

Females (2), dry pinned.

(1, deposited in BPBM). **FIJI**. Taveuni, Cakaudrove Prov., 5.5 km SE Tavuki Vlg., Devo Pk. 1188m, 30.VI–14.VIII.2004 Malaise 1, Schlinger, M. Tokota’a. 16.843°S, 179.966°W. FBA 152624. JBWM Photo 2015-06.

(1, deposited in CNC). **FIJI**. Vanua Levu, Bua Prov., 6 km NW Kilaka, 15.VI–24.VI.2004 Batiqere Range. Malaise. 98m ǁ Schlinger, Tokota’a FJVN58b_M05_07 _16.8067, 178.9914 FBA174462.

#### Diagnosis.

Females of *Paranastatus verticalis* are differentiated by the following combination of features: vertex raised between eyes, and temple flat such that temple and occiput form almost a right angle (Fig. 5); vertex granulate; lower face with fringe of setae below toruli (Fig. 6); mandible tridentate; mesoscutum smooth and convex or flat, not concave (Fig. 16). Males of *Paranastatus verticalis* are differentiated by the following combination of features: vertex granulate; mandible tridentate; in dorsal view occipital margin deeply excavate; colouration darker than that of females.

#### Distribution.

Islands of Fiji, Tonga, Western Samoa (Noyes 2015).

#### Biology.

Parasitoids of *Graeffea crouanii* eggs.

#### Variation.

The two specimens collected in 2004 differ in several features compared to the type series described by Eady (1956). The specimen from Taveuni Island has a body length of 2.85 mm, whereas all specimens in the type series range from 2.4–2.5mm. Unfortunately, an accurate measurement of body length was not possible in the specimen from Vanua Levu because the body is contorted. The scutellar-axillar complex in the new material is dark black-brown (Fig. 18) not light orange-brown (Fig. 16), and the legs are darker than in the type series (Fig. 17). Other slight variations in colour include: temple green-purple laterally, not blue-purple; lower face metallic blue-green, not coppery-green; and pronotum purple-blue dorsally, not purple-coppery. The new material has gastral tergite 1 white, tergite 6 light orange-brown, and remaining tergites dark brown, not gastral tergite 1 white and remaining tergites light brown, or gastral tergite 1 white, tergites 2 and 3 dark brown, and remaining tergites grading to light brown at gastral apex.

### Paranastatus violaceus

Taxon classificationAnimaliaHymenopteraEupelmidae

Masi, 1917

Figs 13 , 25

Paranastatus violaceus Masi, 1917: 166–167.

#### Material examined.

Lectotype female, here designated; dry pinned, deposited in BMNH (Hym Type 5.1,036). Label data: “Silhouette, ’08. Seychelles Exp. Percy Sladen Trust Exped. B.M. 1913-170.”

#### Diagnosis.

Females of *Paranastatus violaceus* are differentiated by the following combination of features: vertex and temple coriaceous; antenna brown except flagellomeres 7 and 8 light yellow-brown (Fig. 25); mandible quadridentate; fore wing evenly infuscate (Fig. 25). Males unknown.

#### Distribution.

Silhouette Island, Seychelles.

#### Biology.

Unknown.

#### Remarks.

Masi (1917) established *Paranastatus violaceus* based on three females, one of which was stated as lacking its gaster. Of the three females, the BMNH only has one complete specimen in its collection (Dale-Skey, pers. comm.). We here designate this female as lectotype and have labelled it accordingly. The location of the other two females is presently unknown.

## Discussion

During the last 100 years, *Paranastatus* has been recorded throughout the South Pacific and from one location in the Indian Ocean (Masi 1917, Eady 1956, Rapp 1995, O’Connor 1955). *Paranastatus egregius* and *Paranastatus violaceus* were described from Seychelles and additional specimens have not been reported since. This may be because these two species are extremely rare, now extinct, or most likely collecting efforts have been insufficient to recover them. Differentiating between these possible reasons would require more intensive sampling of biodiversity, an issue that is important worldwide because of climate change, habitat destruction, and species extinctions. *Paranastatus nigriscutellatus* and *Paranastatus verticalis* have been recorded subsequent to their description, mostly from the islands of Fiji, but also from Tonga (Rapp 1995) and only through rearing rather than collecting. Most of the new material described here was obtained through passive collecting by Malaise traps. This and other passive collecting methods may provide the best way to obtain specimens of *Paranastatus* other than through rearing.

*Graeffea crouanii*, the coconut stick insect, is a pest of coconut palms and is found on many islands throughout the South West Pacific, including Fiji (Deesh et al. 2013). Deesh et al. (2013) hypothesized that *Graeffea crouanii* dispersed in one of three ways: (1) by eggs that fell into canoes from overhanging palms on the beach; (2) by eggs floating across the ocean to other islands because they are saline-tolerant; or (3) simply by the adults being carried on coconut leaves by humans to new locations. The dispersal of eggs of *Graeffea crouanii* could account for the dispersal of *Paranastatus verticalis* and *Paranastatus nigriscutellatus*, suggesting that wherever this stick insect is found, these two species of *Paranastatus* could be found as well.

Gibson (1995) proposed a hypothesis to explain dispersal ability of females in Eupelminae. Because of structural modifications to improve jumping ability, females appear to have reduced flight capabilities that reduce their ability to disperse. He used this hypothesis to explain why better known eupelmine species often have several to numerous hosts, it being advantageous to develop on a wide array of hosts within a limited dispersal area for survival of the parasitoid population. This suggests that species of *Paranastatus* could have a wider host range than is currently recorded.

The current known distribution of *Paranastatus* is perplexing because there are large distances between localities, which leaves the question of how the wasps dispersed through time. One hypothesis that could explain *Paranastatus* distribution is wind dispersal as aerial plankton. Insects have been collected far from land in both the Indian and South Pacific Ocean through aerial netting (Holzapfel and Harrel 1968), and since *Paranastatus* wasps are very small it is possible that they were carried across the ocean on the wind. Another hypothesis is that the wasps dispersed passively through their hosts, such as what may have occurred with parasitized *Graeffea crouanii* eggs (Deesh et al. 2013). If other hosts are discovered similar dispersal mechanisms might also be discovered. It is also possible that the true distribution of this genus has yet to be discovered. Because two of the new species as well as *Paranastatus nigriscutellatus* and *Paranastatus verticalis* are from Fiji, and the other two new species are from Indonesia, it is conceivable that more species of *Paranastatus* exist in other regions of the South Pacific and Indian Ocean.

Another possible explanation for the distribution of *Paranastatus* is that it was once larger than it is now. *Paranastatus* species are basically confined to geographical clusters: the Fijian species have not been found in Indonesia and vice versa. *Paranastatus nigriscutellatus* and *Paranastatus verticalis* have been found on several other South Pacific islands, but only east of Fiji. The two Seychelles species (*Paranastatus egregius* and *Paranastatus violaceus*) have never been found since their original capture. It may be that species of *Paranastatus* did exist in other regions, but have since become extinct. Extinction, if it has happened, may have occurred through habitat fragmentation that reduced the genus to its current number of species and localities. Fragmented habitats can lead to extinction by decreasing available habitat and causing smaller population sizes, and parasitoids tend to be more sensitive to habitat fragmentation than other trophic levels (Kruess and Tscharntke 2000).

It is interesting that the newly collected female specimens of *Paranastatus verticalis* are darker in colour than the original specimens collected in 1954. One possibility for this is that the type specimens have faded over the course of 50 years; however, this does not seem likely as the specimens still conform to Eady’s (1956) description. The two newly collected specimens of *Paranastatus verticalis* are from localities east and west of the original type locality. The slightly more western specimen is from Bua province on Vanua Levu, whereas the eastern specimen is from Cakaudrove province, Taveuni Island. The type specimens were from Savusavu, Vanua Levu, which is centered between Bua province and Taveuni Island. It is possible that continual colour variation would be found across the entire distribution of this species, if sampled, or that females vary in colour pattern based on a specific niche, host, environment or some other difference affecting development.

One complication that arises when studying the taxonomy of Eupelminae is their extreme sexual dimorphism. Non-chalcidologists are likely to identify male eupelmines as Pteromalidae rather than Eupelmidae, and most identification keys to species of eupelmine genera are based only on females, which makes it difficult to correctly identify males unless they are reared together with females. There is a chance that *Paranastatus* males have been collected before, but were misidentified or unidentified. This may account for the lack of males recorded in this genus.

Future work on *Paranastatus* could include a closer examination of the biogeography of the different species. The disparate distribution between *Paranastatus egregius* and *Paranastatus violaceus* from Seychelles, and the remaining species from islands in the South Pacific, suggests that locations between these regions may have additional species of *Paranastatus*. Therefore, it could be worthwhile collecting in regions between Seychelles and Indonesia to improve knowledge of the distribution of the genus or to discover new species.

## Supplementary Material

XML Treatment for Paranastatus bellus

XML Treatment for Paranastatus egregius

XML Treatment for Paranastatus halko

XML Treatment for Paranastatus nigriscutellatus

XML Treatment for Paranastatus parkeri

XML Treatment for Paranastatus pilosus

XML Treatment for Paranastatus verticalis

XML Treatment for Paranastatus violaceus

## Supplementary materials

Supplementary material 1
*Paranastatus* Label Data Data type: Label data Explanation note: This file contains complete label data for all material examined, including localities This dataset is made available under the Open Database License (http://opendatacommons.org/licenses/odbl/1.0/). The Open Database License (ODbL) is a license agreement intended to allow users to freely share, modify, and use this Dataset while maintaining this same freedom for others, provided that the original source and author(s) are credited.Melanie L. Scallion, Gary A.P. Gibson, Barbara J. Sharanowski

## References

[B1] Austin AD Gibson GAP Harvey MS (1998) Synopsis of Australian *Calymmochilus* Masi (Hymenoptera: Eupelmidae), description of a new Western Australian species associated with a pseudoscorpion, and review of pseudoscorpion parasites. Journal of Natural History 32(3): 329–350. doi: 10.1080/00222939800770171

[B2] Deesh AD Swamy BN Khan MGM (2013) Distribution of coconut stick insect, *Graeffea crouanii* and its parasitoids in selected islands of Fiji. Fiji Agriculture Journal 53(1): 18–24.

[B3] Eady RD (1956) Two new species of the genus *Paranastatus* Masi (Hym. Eupelmidae) from Fiji. Bulletin of Entomological Research 47(1): 61–67. doi: 10.1017/S0007485300046514

[B4] Gibson GAP (1989) Phylogeny and classification of Eupelmidae, with a revision of the world genera of Calosotinae and Metapelmatinae (Hymenoptera: Chalcidoidea). Memoirs of the Entomological Society of Canada 121(S149): 3–121. doi: 10.4039/entm121149fv

[B5] Gibson GAP (1995) Parasitic wasps of the subfamily Eupelminae: Classification and revision of world genera (Hymenoptera: Chalcidoidea: Eupelmidae). Memoirs on Entomology, International 5: 1–421.

[B6] Gibson GAP Huber JT Woolley JB (1997) Annotated Keys to the Genera of Nearctic Chalcidoidea (Hymenoptera). NRC Research Press, Ottawa, Canada, 794 pp.

[B7] Harris RA (1979) A glossary of surface sculpturing. Occasional Papers in Entomology 28: 1–31.

[B8] Heraty JM Burks RA Cruaud A Gibson GAP Liljeblad J Munro J Rasplus J-Y Delvare G Janšta P Gumovsky A Huber J Woolley JB Krogmann L Heydon S Polaszek A Schmidt S Darling DC Gates MW Mottern J Murray E Dal Molin A Triapitsyn S Baur H Pinto JD van Noort S George J Yoder M (2013) A phylogenetic analysis of the megadiverse Chalcidoidea (Hymenoptera). Cladistics 29(5): 466–542. doi: 10.1111/cla.12006 10.1111/cla.12006 34798768

[B9] Holzapfel EP Harrell JC (1968) Transoceanic dispersal studies of insects. Pacific Insects 10(1): 115–153.

[B10] Kruess A Tscharntke T (2000) Species richness and parasitism in a fragmented landscape: experiments and field studies with insects on *Vicia sepium*. Oecologia 122(1): 129–137. doi: 10.1007/PL00008829 10.1007/PL00008829 28307950

[B11] Masi L (1917) Chalcididae of the Seychelles Islands. Novitates Zoologicae 24: 121–230.

[B12] Noyes JS (2015) Universal Chalcidoidea Database. http://www.nhm.ac.uk/chalcidoids [accessed 26 June 2015]

[B13] O’Connor BA Pillai JS Singh SR (1955) Notes on the coconut stick insect, *Graeffea crouani* Le Guillou. Fiji Agriculture Journal 25: 89–92.

[B14] Rapp G (1995) Eggs of the stick insect *Graeffea crouanii* Le Guillou (Orthoptera, Phasmidae). Mortality after exposure to natural enemies and high temperature. Journal of Applied Entomology 119(1–5): 89–91. doi: 10.1111/j.1439-0418.1995.tb01249.x

