# Supplementary material for: Revision of Paranastatus Masi (Eupelmidae, Eupelminae) with descriptions of four new species
Source: Zookeys. 2016 Feb 3;(559):59–79. doi: 10.3897/zookeys.559.6134 (PMC4768272; doi:10.3897/zookeys.559.6134)
Supplement: Supplementary material 1 — Paranastatus Label Data [file zookeys-559-059-s001.docx]

*Paranastatus bellus* Scallion, sp. n.

**Holotype (f)**: SULAWESI UTARA: ǁ Dumoga-Bone Nat. Pk. ǁ edge of rainforest, ǁ 0°34’N, 123°54’E. ++ A.D. Austin ǁ June 1985, M.T.

**Paratype (f)**: INDONESIA: Sulawesi ǁ Utara, Dumoga Bone ǁ Nat Pk., Toraut ǁ IV.1985, J.S. Noyes ǁ forest edge, MT

*Paranastatus egregius* Masi, 1917

**Lectotype (f)**: Mahe, ’08-9 ǁ Seychelles Exp. ++ Percy Sladen ǁ Trust Exped. ǁ B.M. 1913-170.

**Paralectotype (m)**: Mahe, ’08–9 ǁ Seychelles Exp. ++ Percy Sladen ǁ Trust Exped. ǁ B.M. 1913-170.

*Paranastatus halko* Scallion, sp. n.

**Holotype (f)**: FIJI: Viti Levu, Vuda Prov., ǁ Koroyanitu Pk., 1 km E Abaca Vlg, ǁ Savuione Trl, 800m, 22.IV-6.V.03 ǁ Malaise 1, Schlinger, Tokota’a., ǁ 17.667°S, 177.55°E. FBA 180165

**Paratypes (24f)**

Fiji: Viti Levu, Vuda Prov., 1 km E ǁ Abaca Vlg., Koroyanitu Ntl. Pk., 800m. ǁ Savuione Trail, 17°40’S, 177°33’E, ǁ 12-19.XI.02, malaise. E. Schlinger ǁ Tokota’a. FJVL01_M01_07. FBA084327

FIJI: Viti Levu, Vuda Prov., ǁ Koroyanitu Pk., 1km E Abaca Vlg, ǁ Savuione Trl, 800m, 22.IV-6.V.03 ǁ Malaise 1, Schlinger, Tokota’a., ǁ 17.667°S, 177.55°E. FBA180167

FIJI: Viti Levu, Naitasiri Prov., ǁ 4 km WSW Colo-i-Suva Village, ǁ Mt. Nakobalevu, 300m, 12.IV.2004, ǁ Malaise 1, Schlinger, Tokota’a. ǁ 18.057°S, 178.42°E. FBA 188657

FIJI: Viti Levu, Naitasiri Prov., ǁ 4 km WSW Colo-i-Suva Village, ǁ Mt. Nakobalevu, 300m, 12.IV.2004, ǁ Malaise 1, Schlinger, Tokota’a. ǁ 18.057°S, 178.42°E. FBA 188656

FIJI: Viti Levu, Vuda Prov. ǁ Koroyanitu Pk., 1km E Abaca Vlg, ǁ 800m, 10-17.XII.2002, Malaise 1, ǁ coll. Schlinger, Tokota’a. ǁ 17.667°S, 177.55°E. FBA185977

FIJI: Viti Levu, Vuda Prov., 1km E ǁ Abaca Vlg., Koroyanitu Ntl. Pk., 800m. ǁ Savuione Trail, 17°40’S, 177°33’E, ǁ 26.X-5.XI.02, malaise. E. Schlinger ǁ Tokota’a. FJVL01_M01_05. FBA082730

FIJI Viti Levu, Vuda Prov. Koroyanitu ǁ Eco Pk., Mt. Evan’s Range, 0.5 km N ǁ Abaca Vlg., [-17.667, 177.55] 800m ǁ 26.XI-3.XII.02, Malaise. Schlinger, ǁ Tokota’a. FJVL03_M01_09. FBA073439 ++ JBWM Photo ǁ 2015-02

FIJI, Viti Levu, Vuda Prov., ǁ Koroyanitu Pk., 1km E Abaca Vlg, ǁ Savuione Trl, 800m, 26.XI-3.XII.02 ǁ Malaise 1, Schlinger Tokota’a., ǁ 17.667°S, 177.55°E, FBA181998

FIJI: Viti Levu, Vuda Prov., ǁ Koroyanitu Pk., 1km E Abaca Vlg, ǁ Savuione Trl, 800m, 22.IV-6.V.03 ǁ Malaise 1, Schlinger Tokota’a., ǁ 17.667°S, 177.55°E, FBA180163

FIJI: Viti Levu, Vuda Prov., ǁ Koroyanitu Pk., 1km E Abaca Vlg, ǁ 800m, 10-17.XII.2002, Malaise 1, ǁ coll. Schlinger Tokota’a. ǁ 17.667°S, 177.55°E. FBA185976

FIJI Viti Levu, Vuda Prov. Koroyanitu ǁ Eco Pk., Mt. Evan’s Range, 0.5 km N ǁ Abaca Vlg., [-17.667, 177.55] 800m ǁ 26.X-5.XI.2002, Malaise. Schlinger, ǁ Tokota’a. FJVL3_M01_05. FBA080116

FIJI: Viti Levu, Vuda Prov., ǁ Koroyanitu Pk., 1kmE Abaca Vlg. ǁ SavuioneTrl,800m, 12-19.X.2002 ǁ Malaise 1, Schlinger Tokota’a, ǁ 17.667°S 177.55°E FBA133407

FIJI, Viti Levu, Vuda Prov., 1km E ǁ Abaca Vlg., Koroyanitu Ntl. Pk., 800m. ǁ Savuione Trail, 17°40’S, 177°33’E, ǁ 26.X-5.XI.02, malaise. E. Schlinger ǁ Tokota’a. FJVL01_M01_05. FBA082728

FIJI, Viti Levu, Vuda Prov., 1km E ǁ Abaca Vlg., Koroyanitu Ntl. Pk., 800m. ǁ Savuione Trail, 17°40’S, 177°33’E, ǁ 7-12.X.02, malaise. E. Schlinger ǁ Tokota’a. FJVL01_M01_02. FBA081475

FIJI, Viti Levu, Vuda Prov., 1km E ǁ Abaca Vlg., Koroyanitu Ntl. Pk., 800m. ǁ Savuione Trail, 17°40’S, 177°33’E, ǁ 12-19.XI.02, malaise. E. Schlinger ǁ Tokota’a. FJVL01_M01_07. FBA084329

FIJI, Viti Levu, Vuda Prov., 1km E ǁ Abaca Vlg., Koroyanitu Ntl. Pk., 800m. ǁ Savuione Trail, 17°40’S, 177°33’E, ǁ 7-12.X.02, malaise. E. Schlinger ǁ Tokota’a. FJVL01_M01_02. FBA081476

FIJI, Viti Levu, Vuda Prov., 1km E ǁ Abaca Vlg., Koroyanitu Ntl. Pk., 800m. ǁ Savuione Trail, 17°40’S, 177°33’E, ǁ 12-19.XI.02, malaise. E. Schlinger ǁ Tokota’a. FJVL01_M01_07. FBA084326

FIJI, Viti Levu, Vuda Prov., 1km E ǁ Abaca Vlg., Koroyanitu Ntl. Pk., 800m. ǁ Savuione Trail, 17°40’S, 177°33’E, ǁ 12-19.XI.02, malaise. E. Schlinger ǁ Tokota’a. FJVL01_M01_07. FBA084328

FIJI Viti Levu, Vuda Prov. Koroyanitu ǁ Eco Pk., Mt. Evan’s Range, 0.5 km N ǁ Abaca Vlg., [-17.667, 177.55] 800m ǁ 26.X-5.XI.2002, Malaise. Schlinger, ǁ Tokota’a. FJVL3_M01_05. FBA080115

FIJI Viti Levu, Vuda Prov. Koroyanitu ǁ Eco Pk., Mt. Evan’s Range, 0.5 km N ǁ Abaca Vlg., [-17.667, 177.55] 800m ǁ 26.X-5.XI.2002, Malaise. Schlinger, ǁ Tokota’a. FJVL3_M01_05. FBA080114

FIJI: Viti Levu, Naitasiri Prov., ǁ 4 km WSW Colo-i-Suva Village, Mt. ǁ Nakobalevu, 372m, 25.II-17.III.03 ǁ Malaise 4, Schlinger, Tokota’a. ǁ 18.055 S, 178.424 E. FBA 103258

FIJI: Viti Levu, Naitasiri Prov., ǁ 4 km WSW Colo-i-Suva Village, Mt. ǁ Nakobalevu, 372m, 25.II-17.III.03 ǁ Malaise 4, Schlinger, Tokota’a. ǁ 18.055 S, 178.424 E. FBA 103259 ++ JBWM Photo ǁ 2015-01

FIJI: Viti Levu, Naitasiri Prov., ǁ 4 km WSW Colo-i-Suva Village, Mt. ǁ Nakobalevu, 372m, 4-14.XI.2003 ǁ Malaise 3, Schlinger, Tokota’a. ǁ 18.055 S, 178.424 E. FBA 096330

FIJI: Viti Levu, Naitasiri Prov., ǁ 4 km WSW Colo-i-Suva Village, Mt. ǁ Nakobalevu, 372m, 4-14.XI.2003 ǁ Malaise 3, Schlinger, Tokota’a. ǁ 18.055 S, 178.424 E. FBA 096331

*Paranastatus nigriscutellatus* Eady, 1956

**Holotype (f)**: HY 976 ǁ FIJI ǁ Savu Savu ǁ ii.1954 ǁ B.A. O’Connor ǁ Ex Graeffea ǁ crouani eggs ++ Com. Inst. Ent ǁ Coll. No. 13599.

**Allotype (m)**: HY 976 ǁ FIJI ǁ Savu Savu II.1954 ǁ B.A. O’Connor ǁ Ex Graeffea ǁ crouani eggs. ++ Com. Inst. Ent ǁ Coll. No. 13599.

**Paratypes (6f)**

HY 975 ǁ FIJI ǁ Taveuni ǁ XI.1953 ǁ B.A. O’Connor ++ Ex eggs of ǁ Graeffea ǁ crouani ǁ in coconut ǁ crowns

HY 976 ǁ FIJI ǁ Savu Savu ǁ ii.1954 ǁ B.A. O’Connor ǁ Ex Graeffea ǁ crouani eggs

HY 975 ǁ FIJI ǁ Taveuni ǁ XI.1953 ǁ B.A. O’Connor ++ Ex eggs of ǁ Graeffea ǁ crouani ǁ in coconut ǁ crowns

HY 976 ǁ FIJI ǁ Savu Savu ǁ ii.1954 ǁ B.A. O’Connor ǁ Ex Graeffea ǁ crouani eggs

HY 976 ǁ FIJI ǁ Savu Savu ǁ ii.1954 ǁ B.A. O’Connor ǁ Ex Graeffea ǁ crouani eggs

HY 975 ǁ FIJI ǁ Taveuni ǁ XI.1953 ǁ B.A. O’Connor ++ Ex eggs of ǁ Graeffea ǁ crouani ǁ in coconut ǁ crowns

**New material (11f)**

FIJI, Viti Levu, Namosi Prov., 2 km SE ǁ Nabukavesi Village, Ocean Pacific Resort, ǁ 40m, 24.XI.-11.XII.2003, Malaise trap: M01, ǁ W. Naisilisili, (-18.171, 178.258). FBA_219645

FIJI: Kaduva I., 0.25 km SW ǁ Solodamu Vlg., Moanakaka Bird ǁ Sanctuary., 60m, 9-30.V.2003 ǁ Malaise 4, Schlinger, Tokota’a. ǁ 19.078°S, 178.121°E. FBA 165997

FIJI: Viti Levu, Vuda Prov., ǁ Koroyanitu Pk., 1 km E Abaca Vlg, ǁ SavuioneTrl, 800m, 22.IV-6.V.03 ǁ Malaise 1, Schlinger, Tokota’a., ǁ 17.667°S 177.55°E FBA 180162

FIJI: Viti Levu, Vuda Prov., ǁ Koroyanitu Pk., 1 km E Abaca Vlg, ǁ SavuioneTrl, 800m, 22.IV-6.V.03 ǁ Malaise 1, Schlinger, Tokota’a., ǁ 17.667°S 177.55°E FBA 180164 ++ JBWM Photo ǁ 2015-03

FIJI, Viti Levu, Namosi Prov., ǁ 2 km SE Nabukavesi Vlg., Ocean ǁ Pacific Rsrt,40m, 13-28.III.2003 ǁ Malaise 1, Schlinger, Tokota’a. ǁ 18.171°S 178.258°E. FBA 182973

FIJI, Viti Levu Isl. Namosi Prov. ǁ 2 km SE Nabukavesi Vlg. Malaise ǁ Ocean Pac. Rsrt. 22.VI-7.VII.03 ǁ Schlinger, Tokota’a FJVL18a_M01_10 ǁ –18.1708, 178.2581 40m FBA051933

FIJI: Viti Levu, Isl. Sigatoka ǁ Prov. Sigatoka Sand Dunes Nat. Pk. ǁ Malaise. 12.ii-12.iii.2003 FJ6B 44m ǁ M. Irwin, E. Schlinger, M. Tokota’a ǁ 18°9’999°S 177°28’910°E FBA024712

FIJI: Levu Is., Maitaesiri Pr. ǁ Hakobalevu Mt., 12-24.iii.03 ǁ 178°25’E 18°03’ 8 rainforest ǁ M. Irwin et al, MT FJ-4D ǁ 340m FBA06468 ++ DNA Voucher ǁ D # 2322 ǁ UCR, J.M. Heraty

FIJI, Viti Levu, Namosi Prov., 2 km SE ǁ Nabukavesi Village, Ocean Pacific Resort, ǁ 40m, 24.XI.-11.XII.2003, Malaise trap: M01, ǁ W. Naisilisili, (-18.171, 178.258). FBA_219643

FIJI : Viti Levu, Naitasiri Prov., ǁ Bakobalevu logging road. ǁ 17.III.-9.IV.2003. Malaise Trap: ǁ M01. E.I. Schlinger. M. Tokotaa. ǁ FBA211330

FIJI, Viti Levu, Namosi Prov., 2 km SE ǁ Nabukavesi Village, Ocean Pacific Resort, ǁ 40m, 24.XI.-11.XII.2003, Malaise trap: M01, ǁ W. Naisilisili, (-18.171, 178.258). FBA_219646

*Paranastatus parkeri* Scallion, sp. n.

**Holotype (f)**: FIJI: Viti Levu, 3.5 km N Veisari ǁ Stlmt., logging rd. to Waivudawa, ǁ 14.II-8.III.03, 300m, Malaise 3, ǁ coll. E. Sclinger, M. Tokota’a ǁ 18.068°S, 178.367°E. FBA 136331

*Paranastatus pilosus* Scallion, sp. n.

**Holotype (f)**: INDONESIA ǁ Seram, Solea ǁ VIII. 1987, MT ǁ M. Day, forest

**Paratypes (8f)**

INDONESIA ǁ Seram, Solea ǁ IX 1987 ǁ M. Day ++ JBWM Photo ǁ 2015-05

INDONESIA ǁ Seram, Solea ǁ VIII. 1987 ǁ M. Day

INDONESIA ǁ Seram, Solea ǁ IX 1987 ǁ M. Day

INDONESIA ǁ Seram, Solea ǁ IX 1987 ǁ M. Day

INDONESIA ǁ Seram, Solea ǁ VIII. 1987, MT ǁ M. Day, forest

INDONESIA ǁ Seram, Solea ǁ IX 1987 ǁ M. Day

INDONESIA ǁ Seram, Solea ǁ VIII. 1987, MT ǁ M. Day, forest

INDONESIA ǁ Seram, Solea ǁ VIII. 1987, MT ǁ M. Day, forest

*Paranastatus verticalis* Eady, 1956

**Holotype (f)**: HY 976 ǁ FIJI ǁ Suva Suva ǁ vi.1954 ǁ B.A. O’Connor ++ ex eggs of ǁ Graeffea crouani ǁ C.I.E.Coll. 13792

**Allotype (m)**: HWY 976 ǁ FIJI ǁ Suva Suva ǁ VI.1954 ǁ B.A. O’Connor. ++ ex eggs of ǁ Graeffea crouani ǁ C.I.E.Coll. 13792.

**Paratypes (7f)**

HY 976 ǁ FIJI ǁ Suva Suva ǁ vi.1954 ǁ B.A. O’Connor ++ ex eggs of ǁ Graeffea crouani ǁ C.I.E.Coll. 13792 ++ JBWM Photo ǁ 2015-04

HY 976 ǁ FIJI ǁ Suva Suva ǁ vi.1954 ǁ B.A. O’Connor ++ ex eggs of ǁ Graeffea crouani ǁ C.I.E.Coll. 13792

HY 976 ǁ FIJI ǁ Suva Suva ǁ vi.1954 ǁ B.A. O’Connor ++ ex eggs of ǁ Graeffea crouani ǁ C.I.E.Coll. 13792

HY 976 ǁ FIJI ǁ Suva Suva ǁ vi.1954 ǁ B.A. O’Connor ++ ex eggs of ǁ Graeffea crouani ǁ C.I.E.Coll. 13792

HY 976 ǁ FIJI ǁ Suva Suva ǁ vi.1954 ǁ B.A. O’Connor ++ ex eggs of ǁ Graeffea crouani ǁ C.I.E.Coll. 13792

HY 976 ǁ FIJI ǁ Suva Suva ǁ vi.1954 ǁ B.A. O’Connor ++ ex eggs of ǁ Graeffea crouani ǁ C.I.E.Coll. 13792

HY 976 ǁ FIJI ǁ Suva Suva ǁ vi.1954 ǁ B.A. O’Connor ++ ex eggs of ǁ Graeffea crouani ǁ C.I.E.Coll. 13792

**New material (2f)**

FIJI: Taveuni, Cakaudrove Prov., ǁ 5.5 km SE Tavuki Vlg., Devo Peak, ǁ 1188m, 30.VI-14.VIII.2004, ǁ Malaise 1, Schlinger, M. Tokota’a. ǁ 16.843°S, 179.966°W. FBA 152624 ++ JBWM Photo ǁ 2015-06

FIJI. Vanua Levu Island. Bua Prov. ǁ 6 km NW Kilaka. 15.VI-24.VI.04 ǁ Batiqere Range. Malaise. 98m ǁ Schlinger, Tokota’a FJVN58b_M05_07 ǁ _16.8067, 178.9914 FBA174462

*Paranastatus violaceus* Masi, 1917

**Lectotype (f)**: Silhouette, ’08. ǁ Seychelles Exp. ++ Percy Sladen ǁ Trust Exped. ǁ B.M. 1913-170.
